# Supplementary material for: Individualised Nutritional Care for Disease-Related Malnutrition: Improving Outcomes by Focusing on What Matters to Patients
Source: Nutrients. 2022 Aug 27;14(17):3534. doi: 10.3390/nu14173534 (PMC9460401; doi:10.3390/nu14173534)
Supplement: Supplementary file 1 [file nutrients-14-03534-s001.zip › nutrients-1860079-supplementary.pdf]

Table S1. Individualised nutritional care in nutrition guidelines.

Title, reference	Organisation, resource type, year	Extracted statements, recommendations or standards relating to aspects of individualised nutritional care as expressed in the original source publication
**General – relating to provision of nutrition support across multiple patient groups/diseases/conditions**		
Home enteral nutrition [1]	European Society for Clinical Nutrition and Metabolism (ESPEN) Practical Guideline 2022	3.2.4. Administration<br>3.2.4.1. Nutrition support team<br>30) The method of HEN administration should be a decision of the multidisciplinary NST involved with the patient care, considering patient's disease, type of feeding tube in position, feed tolerance and patient preference.
Hospital nutrition [2]	ESPEN Guideline 2021	NOT PART OF RECOMMENDATIONS BUT MENTIONED IN THE COMMENTARY<br>Nutritional needs should be assessed individually for every patient including considering nutritional status, physical activity level, disease status and tolerance, length of hospitalization, and chronic disease.<br>Individual adaptation must be proposed according to food preferences and levels of food intake.<br>4.3. Which could be the standard of logistics for hospital food delivery?<br>4.3.3. Recommendation 20<br>Hospital food delivery must be adapted to patient's abilities and perspectives (acute care, rehabilitation unit, palliative care). Grade of Recommendation GPP e strong consensus (92.3% agreement).<br>Commentary. Patient's needs and capabilities depend on clinic situations and patient perspectives. Hospital food should be adapted accordingly. For palliative care, the organization of food provision is part of nutritional care as well as a comprehensive approach to nutritional care and individualization of nutrition<br>4.3.4. Recommendation 21<br>Commentary. Hospital food delivery should be different regarding the patient's abilities, type of hospitalization, and perspectives. Meals should meet patient's preferences and abilities to eat.<br>4.4.2. Should vegan diet, religious beliefs, food preferences, presumed food intolerance, beliefs be taken into account for the composition of the standard diet?<br>4.4.2.1. Recommendation 23. Religious beliefs and food preferences (taste) should be taken into account at best when proposing the menu choice to the patient.

		4.5.2. What are the indications for a high-energy diet and/or high protein diet?<br>4.5.2.1. Recommendation 29. Commentary. Geriatric patients (at least 1 g protein/kg BW/day. The amount should be individually adjusted concerning nutritional status, physical activity level, disease status, and tolerance)<br>4.5.2.2. Recommendation 30. Commentary. Ideally, a combination of a specifically designed high energy high protein diet, snacking, ONS, and nutritional counseling should be available in the acute hospital setting to provide the most individualized nutrition therapy.<br>4.7.2. Is there a recommended diet for diabetic patients?<br>4.7.2.3. Recommendation 45. Snacks containing mixed carbohydrates and protein should be offered between meals according to individual care (e.g. usually with mealtime short- and median acting insulin) and glycemic control.<br>4.7.2.5. Recommendation 47. With diabetic complications (e.g. diabetic nephropathy, diabetic gastroparesis, lower limb ulcers, and amputations), diet and nutrition support should be individual and diagnosis-based.
Ethical aspects of artificially administered nutrition and hydration [3]	American Society for Parenteral and Enteral Nutrition (ASPEN) Position Paper 2021	ASPEN Position Statements:<br>• The 4 ethical principles of autonomy, beneficence, nonmaleficence, and justice should be equally applied to patient care[1].<br>• The cultural values, religious beliefs, ethnic background, country, region, and geographical considerations of patients and families need to be respected to the extent that they are consistent with the ethical principles and duties and legal requirements.<br>• For individuals with cancer, use of a patient-centered communication style—if the patient, family, or surrogate decision maker desires—incorporates a shared decision-making process. Conflicts between the clinician obligations and the patient's preferences involving AANH use should be acknowledged and evaluated on a case-by-case basis.<br>• For persons at the end of life (EOL), their preferences/QOL goals with acceptance or refusal of modified-consistency food and fluids provided orally or AANH must be respected.
Home parenteral nutrition [4]	ESPEN Guideline 2020	3. What are the criteria for a safe HPN program?<br>Commentary. The HPN program shall provide an individualized, safe, effective and appropriate nutrition support plan at discharge from hospital which should then be supervised and evaluated on a regular basis in the community<br>7. Which nutritional PN admixture bag should be chosen?<br>Recommendation 39

		Either commercially available ready-to-use admixtures or customized and tailored to the individual patient's requirements admixtures can be used for HPN.<br>Recommendation 40<br>Customized and tailored HPN admixtures can be prepared either by individual compounding or by ready-to-use prepared and adapted commercial multi-chamber bags, according to the manufacturer instructions and using aseptic admixture technique preferably in a laminar flow cabinet.<br>12. Which are the local and personnel preconditions for HPN?<br>Recommendation 58<br>A formal individualized HPN training program for the patient and/or caregiver and/or home care nurses shall be performed, including catheter care, pump use and preventing, recognizing and managing complications; training can be done in an in-patient setting or at the patient's home.
Selection and care of central venous access devices for adult home parenteral nutrition administration [5]	ASPEN Guideline 2019	Introduction:<br>• The recommendations provided in this guideline are tailored to address these issues and provide a science-based starting point for individualized HPN therapy.<br>Target Patient Population for Guidelines<br>• These guidelines are directed toward generalized outpatient populations but, like any other management strategy, the infusion therapy selected should be tailored to the individual patient.
Nutrition support: adult hospitalized patients[6]	ASPEN Standard 2018	Standard 7. Interprofessional Approach:<br>• The nutrition care plan should be developed using an interprofessional team approach involving the patient, caregiver (if applicable), the nutrition support service (or team), the patient’s physician(s), dietitian(s), nurse(s), pharmacist(s), and other appropriate healthcare professionals.<br>Standard 8. Patient and Caregiver Communication:<br>• The nutrition care plan should include patient and/or caregiver(s) education about nutrition support therapy, goals, and expectations and should incorporate the wishes of the patients and/or caregiver(s). Appropriate routes of administration shall be defined, identification of intake goals shall be included, and estimated duration of therapy as well as criteria for discontinuation of therapy should be addressed.<br>Standard 12. Nutrition Support Access:<br>• The selection of a venous access site (central vs peripheral vein) should depend on expected duration of therapy, nutrition requirements, and patient’s vascular condition and preferences.<br>• The selection of an enteral access device (nasoenteric vs enterostomy [ie, gastrostomy, jejunostomy]) should depend on the patient’s disease state, needs and goals, ethical situation, gastrointestinal

		anatomy and function, expected duration of EN therapy, and the ability to safely access the gastrointestinal tract via radiologic, surgical, endoscopic techniques, or other guided technology.
Safe practices for enteral nutrition therapy [7]	ASPEN Consensus Recommendations 2017	Question 6.3. What are the essential steps in EN administration to prevent aspiration?<br>• Understand that the method of administration (bolus, intermittent, continuous) and optimal site (gastric, small bowel) of EN feeding will depend on the patient needs, medical conditions, tolerance and goals (e.g, if home use is anticipated), and resources available.<br>Question 6.7. What factors determine the best duration or rate of the feeding to improve the likelihood that the full prescribed dose is received?<br>• Consider patient condition factors and tolerance, lifestyle, goals and convenience, and placement of the distal end of the tube in formulating the feeding regimen to meet patient nutrition and fluid needs.<br>Question 6.10. Under what circumstances (if any) should EN be held to improve patient safety (prior to transportation, prior to procedures, surgery, or extubation)?<br>• Consider risk vs benefit regarding disconnection of EN on an individual basis as it reduces needed nutrient delivery and may increase safety risk<br>Question 10.3. What is the best way to transition from EN to oral feeding?<br>• Identify a safe oral feeding regimen through discussion with interdisciplinary team members, including speech and language specialists who evaluate swallowing and aspiration risk with various food consistencies. Provide an individualized diet with necessary modifications in the initial stages of oral intake. Involve the patient and/or family members in food and oral supplement preferences regarding oral diet advancement.<br>The recommendations within this document are intended for discussion and adoption over time by organizations involved in the delivery of EN. These recommendations are not intended to supersede the judgment of the healthcare professional or employing institution based on the circumstances of the individual patient
**Intensive care**		
Provision of nutrition support therapy in the adult critically ill patient [8]	ASPEN Guideline 2021	Guideline question 2:<br>• In adult critically ill patients, does provision of higher as compared with lower protein intake impact clinical outcomes?<br>• GRADE recommendation: There was no difference in clinical outcomes in the relatively limited data. Because of a paucity of trials with high-quality evidence, we cannot make a new recommendation at this time beyond the 2016 guideline suggestion for 1.2–2.0 g/kg/day.

		• Discussion on clinical application for question 2: Few studies have investigated the impact of higher protein doses provided with equivalent energy; thus, the impact on outcomes is not known. Until more data are available, we suggest clinicians should individualize protein prescriptions based on clinician judgment of estimated needs.
Clinical nutrition in the intensive care unit [9]	ESPEN Guideline 2019	Introduction<br>• The large heterogeneity of the ICU population potentially reduces the external validity of the recommendations, which should be seen as a basis to support decisions made for each patient on an individual basis
**Covid-19**		
Nutritional management of individuals with obesity and COVID-19 [10]	ESPEN expert statements and practical guidance 2021	c) Nutritional management of persons with obesity for prevention of SARS-CoV-2 infection and potential poor COVID-19 outcomes<br>Exercise<br>Potential health- and performance status related contraindications or limitations for exercise should be assessed before the initiation of activity or exercise programmes, and individualized recommendations should be provided as much as possible with regard to exercise type, frequency, intensity and duration by experienced healthcare professionals<br>d) Nutritional management of persons with obesity and COVID-19<br>Non-ICU hospital setting<br>Included in commentary: Importantly, nutritional treatment should continue also after discharge from the hospital with individualized nutritional plans, in order to prevent or limit the likely further worsening of nutritional status, which may result from pre-existing derangements and acute disease- and hospitalization-induced catabolic stimuli.<br>Intubated patients<br>Similar to non-ICU settings, mobilization and physical activity when possible should be implemented with structured individualized protocols aimed at enhancing protein anabolism<br>f) Nutritional management of persons with obesity during recovery from COVID-19<br>19. Due to high risk of loss of skeletal muscle mass with ensuing malnutrition and sarcopenia, persons with obesity recovering from COVID-19 should be encouraged to perform safe and adequate physical activity including exercise programmes, particularly in the presence of older age, comorbidities or previous ICU stay, ideally within individualized programmes established by experienced professionals.
Nutritional management of individuals with	ESPEN	2. Prevention and treatment of malnutrition in individuals at risk or infected with SARS-CoV-2<br>2.2. Statement 2

SARS-CoV-2 infection [11]	Expert statements and practical guidance 2020	Energy needs can be assessed using indirect calorimetry if safely available with ensured sterility of the measurement system, or as alternatives by prediction equations or weight-based formulae such as:<br>30 kcal per kg body weight and day; guiding value for energy intake in older persons, this value should be individually adjusted with regard to nutritional status, physical activity level, disease status and tolerance.<br>Protein needs are usually estimated using formulae such as:<br>1 g protein per kg body weight and day in older persons; the amount should be individually adjusted with regard to nutritional status, physical activity level, disease status and tolerance<br>2.5. Statement 5<br>Comments under statement 5 - Nutritional treatment should continue after hospital discharge with ONS and individualized nutritional plans; this is particularly important since pre-existing nutritional risk factors continue to apply and acute disease and hospitalization are likely to worsen the risk or condition of malnutrition.
Nutrition management for critically and acutely unwell hospitalised patients with coronavirus disease 2019 (COVID-19) in Australia and New Zealand [12]	Guideline 2020	Nutrition care should be tailored to pandemic capacity, with early gastric feeding commenced using an algorithm to provide nutrition for the first 5–7 days in lower-nutritional-risk patients and individualised care for high-nutritional-risk patients where capacity allows.
**Cancer**		
Clinical Nutrition in cancer [13]	ESPEN Practical Guideline 2021	3. General concepts of treatment relevant to all cancer patients<br>3.2. Energy and substrate requirements<br>We recommend that the total energy expenditure (TEE) of cancer patients, if not measured individually, be assumed to be similar to healthy subjects and generally ranging between 25 and 30 kcal/kg/day.<br>3.4. Exercise<br>13) We suggest individualized resistance exercise in addition to aerobic exercise to maintain muscle strength and muscle mass.<br>5. Interventions relevant to specific patient categories<br>5.2. Radiotherapy

		We recommend that during radiotherapy e with special attention to radiotherapy of the head and neck, thorax and GI tract - an adequate nutritional intake should be ensured primarily by individualized nutritional counseling and/or with use of ONS, in order to avoid nutritional deterioration, maintain intake and avoid radiotherapy interruptions.
Cancer cachexia in adult patients [14]	The European Society For Medical Oncology (ESMO) Clinical Practice Guidelines 2021	Screening and assessment of nutritional and metabolic risk for cachexia.<br>[after comprehensive assessment by a nutrition expert] Start a tailored intervention: Individualised nutritional intervention by a nutritionally-trained professional team, alleviation/treatment of nutrition impact symptoms, psychological/social support, (supervised) physical exercise (strength, endurance), consider anticancer treatment.<br>Choosing anti-cachexia treatment options: prioritising multimodal care<br>Given the complex and multifaceted contributors to cachexia, anti-cachexia treatment must be based on a comprehensive assessment of the patient's situation and an evaluation of reasonable, available treatment options, resulting in a personalised, multitargeted and multimodal approach.<br>Nutritional support and physiotherapy may be offered on an individual basis while carefully monitoring individual goals and QoL.<br>Tube feeding<br>It is critical to recognise the emergence of dysphagia early and to respond in a timely and individually appropriate way to safeguard adequate feeding. This may include diagnostic procedures to classify and grade swallowing deficits, involving a speech therapist, specialised dietary counselling and products either via nasogastric tube feeding (NTF) or percutaneous tube feeding [e.g. percutaneous endoscopic gastrostomy (PEG)].<br>Given the lack of reliable clinical evidence of superior outcome for either method, it has been proposed that patient preference be considered when deciding on the feeding method<br>Parenteral Nutrition<br>The decision to initiate PN should be individualised based on the extent of disease, physical and psychological resources of the patient and on a case-by-case risk/benefit assessment.
Cancer-related malnutrition and sarcopenia [15]	The Clinical Oncology Society of Australia (COSA) Position Statement 2020	3.1 Identifying cancer-related malnutrition and sarcopenia<br>3.3 Treating cancer-related malnutrition and sarcopenia<br>1. All people with cancer-related malnutrition and sarcopenia should have access to the core components of treatment including individualised medical nutrition therapy, targeted exercise prescription and physical activity advice and physical and psychological symptom management.

		2. Treatment for cancer-related malnutrition and sarcopenia should be individualised, in collaboration with the multidisciplinary team (MDT), and tailored to consider multi-morbidities and meet needs at each stage of cancer treatment.<br>3.4 Role of the multidisciplinary team<br>2. MDTs should work towards an individualised and coordinated approach to treating cancer-related malnutrition and sarcopenia.
**Older people**		
Clinical nutrition and hydration in geriatrics [16]	ESPEN Guideline 2019	1.3. Ethical aspects regarding nutritional interventions in older persons<br>In all cases, respecting the patient's will and preferences is of utmost priority<br>I. Basic questions and general principles<br>I.1 How much energy and nutrients should be offered/delivered to older persons?<br>Recommendation 1<br>Guiding value for energy intake in older persons is 30 kcal per kg body weight and day; this value should be individually adjusted with regard to nutritional status, physical activity level, disease status and tolerance.<br>Recommendation 2<br>Protein intake in older persons should be at least 1 g protein per kg body weight and day. The amount should be individually adjusted with regard to nutritional status, physical activity level, disease status and tolerance.<br>I.2 How should nutritional care be organized in older persons?<br>Recommendation 6<br>A positive malnutrition screening shall be followed by systematic assessment, individualized intervention, monitoring and corresponding adjustment of interventions.<br>I.3 How should nutritional care be performed in older persons?<br>Recommendation 8<br>Nutritional and hydration care for older persons shall be individualized and comprehensive in order to ensure adequate nutritional intake, maintain or improve nutritional status and improve clinical course and quality of life.<br>Commentary: Nutritional problems are multifaceted and differ between individuals. Moreover, older persons are heterogeneous regarding health status, prognosis, physiological resources, nutritional needs, preferences, and individual goals. In this light it seems reasonable to adapt nutritional interventions individually.

		II.3 Should older persons with malnutrition or at risk of malnutrition be encouraged to share their mealtimes with others?<br>Recommendation 14<br>Commentary: As for all other interventions, here also decisions shall always be individualized according to the persons needs and preferences.<br>Nutritional counseling<br>II.7 Should older persons with malnutrition or at risk of malnutrition be offered individualized nutritional counseling?<br>Recommendation 18<br>Older persons with malnutrition or at risk of malnutrition and/or their caregivers should be offered individualized nutritional counseling in order to support adequate dietary intake and improve or maintain nutritional status.<br>Recommendation 19<br>Individualized nutritional counseling should be offered by a qualified dietician to these persons and/or their caregivers, should consist of several (at least 2) individual sessions that may be combined with group sessions, telephone contacts and written advice and should be maintained over a longer period of time.<br>Enteral and parenteral nutrition<br>II.12 Should enteral tube feeding be offered to older persons with malnutrition or at risk of malnutrition?<br>Recommendation 30<br>The expected benefits and potential risks of EN shall be evaluated individually and reassessed regularly and when the clinical condition changes.<br>III. Recommendations for older persons with specific diseases<br>III.1 Should older patients after hip fracture and orthopedic surgery be offered nutritional support?<br>Recommendation 46<br>Nutritional interventions in geriatric patients after hip fracture and orthopedic surgery shall be part of an individually tailored, multidimensional and multidisciplinary team intervention in order to ensure adequate dietary intake, improve clinical outcomes and maintain quality of life.
Individualized nutrition approaches for older adults: long-term care, post-acute	The Academy of Nutrition and Dietetics Position	The Academy advocates that as part of the interprofessional team, registered dietitian nutritionists assess, evaluate, and recommend appropriate nutrition interventions according to each individual's medical<br>condition, desires, and rights to make health care choices.

care, and other settings [17]	Paper 2018

[1](1) autonomy, respect the patient's healthcare preferences; (2) beneficence, provide healthcare in the best interest of the patient; (3) nonmaleficence, do no harm; and (4) justice, provide all individuals a fair and appropriate distribution of healthcare resources.

References

1. Bischoff, S.C.; Austin, P.; Boeykens, K.; Chourdakis, M.; Cuerda, C.; Jonkers-Schuitema, C.; Lichota, M.; Nyulasi, I.; Schneider, S.M.; Stanga, Z., et al. ESPEN practical guideline: Home enteral nutrition. *Clinical Nutrition* **2022**, *41*, 468-488, doi:10.1016/j.clnu.2021.10.018.
2. Thibault, R.; Abbasoglu, O.; Ioannou, E.; Meija, L.; Ottens-Oussoren, K.; Pichard, C.; Rothenberg, E.; Rubin, D.; Siljamäki-Ojansuu, U.; Vaillant, M.-F., et al. ESPEN guideline on hospital nutrition. *Clinical Nutrition* **2021**, *40*, 5684-5709, doi:10.1016/j.clnu.2021.09.039.
3. Schwartz, D.B.; Barrocas, A.; Annetta, M.G.; Stratton, K.; McGinnis, C.; Hardy, G.; Wong, T.; Arenas, D.; Turon-Findley, M.P.; Kliger, R.G., et al. Ethical Aspects of Artificially Administered Nutrition and Hydration: An ASPEN Position Paper. *Nutrition in Clinical Practice* **2021**, *36*, 254-267, doi:https://doi.org/10.1002/ncp.10633.
4. Pironi, L.; Boeykens, K.; Bozzetti, F.; Joly, F.; Klek, S.; Lal, S.; Lichota, M.; Mühlebach, S.; Van Gossum, A.; Wanten, G., et al. ESPEN guideline on home parenteral nutrition. *Clinical Nutrition* **2020**, *39*, 1645-1666, doi:10.1016/j.clnu.2020.03.005.
5. Kovacevich, D.S.; Corrigan, M.; Ross, V.M.; McKeever, L.; Hall, A.M.; Braunschweig, C. American Society for Parenteral and Enteral Nutrition Guidelines for the Selection and Care of Central Venous Access Devices for Adult Home Parenteral Nutrition Administration. *JPEN. Journal of parenteral and enteral nutrition* **2019**, *43*, 15-31, doi:10.1002/jpen.1455.
6. Ukleja, A.; Gilbert, K.; Mogensen, K.M.; Walker, R.; Ward, C.T.; Ybarra, J.; Holcombe, B.; Task Force on Standards for Nutrition Support: Adult Hospitalized Patients, t.A.S.f.P.; Nutrition, E. Standards for Nutrition Support: Adult Hospitalized Patients. *Nutrition in Clinical Practice* **2018**, *33*, 906-920, doi:https://doi.org/10.1002/ncp.10204.
7. Boullata, J.I.; Carrera, A.L.; Harvey, L.; Escuro, A.A.; Hudson, L.; Mays, A.; McGinnis, C.; Wessel, J.J.; Bajpai, S.; Beebe, M.L., et al. ASPEN Safe Practices for Enteral Nutrition Therapy. *Journal of Parenteral and Enteral Nutrition* **2017**, *41*, 0148607116673053, doi:https://doi.org/10.1177/0148607116673053.
8. Compher, C.; Bingham, A.L.; McCall, M.; Patel, J.; Rice, T.W.; Braunschweig, C.; McKeever, L. Guidelines for the provision of nutrition support therapy in the adult critically ill patient: The American Society for Parenteral and Enteral Nutrition. *JPEN. Journal of parenteral and enteral nutrition* **2021**, *n/a*, doi:10.1002/jpen.2267.
9. Singer, P.; Blaser, A.R.; Berger, M.M.; Alhazzani, W.; Calder, P.C.; Casaer, M.P.; Hiesmayr, M.; Mayer, K.; Montejo, J.C.; Pichard, C., et al. ESPEN guideline on clinical nutrition in the intensive care unit. *Clin Nutr* **2019**, *38*, 48-79, doi:10.1016/j.clnu.2018.08.037.
10. Barazzoni, R.; Bischoff, S.C.; Busetto, L.; Cederholm, T.; Chourdakis, M.; Cuerda, C.; Delzenne, N.; Genton, L.; Schneider, S.; Singer, P., et al. Nutritional management of individuals with obesity and COVID-19: ESPEN expert statements and practical guidance. *Clin Nutr* **2021**, 10.1016/j.clnu.2021.05.006, doi:10.1016/j.clnu.2021.05.006.

11. Barazzoni, R.; Bischoff, S.C.; Breda, J.; Wickramasinghe, K.; Krznaric, Z.; Nitzan, D.; Pirlich, M.; Singer, P.; endorsed by the, E.C. ESPEN expert statements and practical guidance for nutritional management of individuals with SARS-CoV-2 infection. *Clin Nutr* **2020**, *39*, 1631-1638, doi:10.1016/j.clnu.2020.03.022.
12. Chapple, L.S.; Fetterplace, K.; Asrani, V.; Burrell, A.; Cheng, A.C.; Collins, P.; Doola, R.; Ferrie, S.; Marshall, A.P.; Ridley, E.J. Nutrition management for critically and acutely unwell hospitalised patients with coronavirus disease 2019 (COVID-19) in Australia and New Zealand. *Nutr Diet* **2020**, *77*, 426-436, doi:10.1111/1747-0080.12636.
13. Muscaritoli, M.; Arends, J.; Bachmann, P.; Baracos, V.; Barthelemy, N.; Bertz, H.; Bozzetti, F.; Hutterer, E.; Isenring, E.; Kaasa, S., et al. ESPEN practical guideline: Clinical Nutrition in cancer. *Clin Nutr* **2021**, *40*, 2898-2913, doi:10.1016/j.clnu.2021.02.005.
14. Arends, J.; Strasser, F.; Gonella, S.; Solheim, T.S.; Madeddu, C.; Ravasco, P.; Buonaccorso, L.; de van der Schueren, M.A.E.; Baldwin, C.; Chasen, M., et al. Cancer cachexia in adult patients: ESMO Clinical Practice Guidelines[☆]. *ESMO Open* **2021**, *6*, doi:10.1016/j.esmoop.2021.100092.
15. Kiss, N.; Loeliger, J.; Findlay, M.; Isenring, E.; Baguley, B.J.; Boltong, A.; Butler, A.; Deftereos, I.; Eisenhuth, M.; Fraser, S.F., et al. Clinical Oncology Society of Australia: Position statement on cancer-related malnutrition and sarcopenia. *Nutr Diet* **2020**, *77*, 416-425, doi:10.1111/1747-0080.12631.
16. Volkert, D.; Beck, A.M.; Cederholm, T.; Cruz-Jentoft, A.; Goisser, S.; Hooper, L.; Kiesswetter, E.; Maggio, M.; Raynaud-Simon, A.; Sieber, C.C., et al. ESPEN guideline on clinical nutrition and hydration in geriatrics. *Clin Nutr* **2019**, *38*, 10-47, doi:10.1016/j.clnu.2018.05.024.
17. Dorner, B.; Friedrich, E.K. Position of the Academy of Nutrition and Dietetics: Individualized Nutrition Approaches for Older Adults: Long-Term Care, Post-Acute Care, and Other Settings. *Journal of the Academy of Nutrition and Dietetics* **2018**, *118*, 724-735, doi:10.1016/j.jand.2018.01.022.
